# 5-Methyl-1-phenyl-1*H*-1,2,3-triazole-4-carboxylic acid

**DOI:** 10.1107/S1600536808027311

**Published:** 2008-08-30

**Authors:** Jin Rui Lin, Ji Yuan Yao, Hong Zhao

**Affiliations:** a Ordered Matter Science Research Center, College of Chemistry and Chemical Engineering, Southeast University, Nanjing 210096, People’s Republic of China

## Abstract

The title compound, C_10_H_9_N_3_O_2_, was synthesized from azido­benzene and ethyl acetyl­acetate. A pair of hydrogen bonds [2.617 (2) Å] inter­connects a pair of the carboxyl groups, forming an *R* ^2^_2_(8) inversion dimer, a frequent motif in carboxylic acids. In the title structure, the bonding H atom in the aforementioned O—H⋯O hydrogen bond is significantly shifted towards the acceptor O atom [the donor and acceptor O—H distances are 1.25 (4) and 1.38 (4) Å, respectively]. A plot of the O⋯O *versus* O—H distances in compounds with paired carboxyl groups shows that the title structure belongs to the group of structures with abnormally long O—H distances with regard to the O⋯O contacts. The displacement of the bonding H atom towards the centre of the hydrogen bond is concomitant with more equal C—O bonding distances in the carboxyl group.

## Related literature

For related literature, see: El Khadem *et al.* (1968 ▶); Olesen *et al.* (2003 ▶); Tian *et al.* (2005 ▶); Allen (2002 ▶); Etter *et al.* (1990 ▶); Radl *et al.* (2000 ▶).
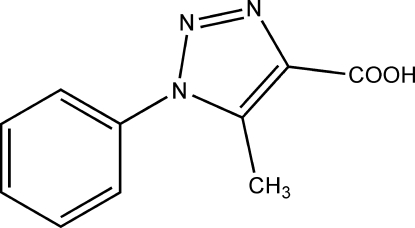


## Experimental

### 

#### Crystal data

C_10_H_9_N_3_O_2_
*M* *_r_* = 203.20
Monoclinic, 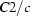

*a* = 23.616 (3) Å
*b* = 7.7189 (15) Å
*c* = 12.606 (2) Å
β = 113.18 (3)°
*V* = 2112.5 (8) Å^3^
*Z* = 8
Mo *K*α radiation
μ = 0.09 mm^−1^
*T* = 293 (2) K
0.20 × 0.18 × 0.15 mm

#### Data collection

Rigaku SCXmini diffractometer
Absorption correction: multi-scan (*CrystalClear*; Rigaku, 2005 ▶) *T*_min_ = 0.965, *T*_max_ = 0.977
10370 measured reflections
2400 independent reflections
1583 reflections with *I* > 2σ(*I*)
*R*_int_ = 0.053

#### Refinement

*R*[*F* ^2^ > 2σ(*F* ^2^)] = 0.062
*wR*(*F* ^2^) = 0.148
*S* = 1.08
2400 reflections
141 parameters
H atoms treated by a mixture of independent and constrained refinement
Δρ_max_ = 0.14 e Å^−3^
Δρ_min_ = −0.18 e Å^−3^

### 

Data collection: *CrystalClear* (Rigaku, 2005 ▶); cell refinement: *CrystalClear*; data reduction: *CrystalClear*; program(s) used to solve structure: *SHELXS97* (Sheldrick, 2008 ▶); program(s) used to refine structure: *SHELXL97* (Sheldrick, 2008 ▶); molecular graphics: *SHELXTL/PC* (Sheldrick, 2008 ▶); software used to prepare material for publication: *SHELXTL/PC*.

## Supplementary Material

Crystal structure: contains datablocks I, global. DOI: 10.1107/S1600536808027311/fb2106sup1.cif

Structure factors: contains datablocks I. DOI: 10.1107/S1600536808027311/fb2106Isup2.hkl

Additional supplementary materials:  crystallographic information; 3D view; checkCIF report

## Figures and Tables

**Table 1:** Hydrogen-bond geometry (Å, °)

*D*—H⋯*A*	*D*—H	H⋯*A*	*D*⋯*A*	*D*—H⋯*A*
O2—H2⋯O1^i^	1.25 (4)	1.38 (4)	2.617 (2)	173 (3)

Symmetry code: (i) 
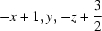
.

**Fig. 1.**
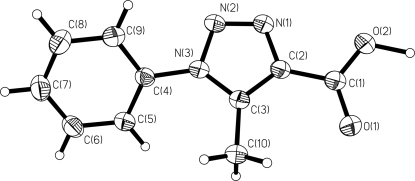
The title molecule, showing the atomic numbering scheme. The displacement ellipsoids are drawn at the 50% probability level.

**Fig. 2.**
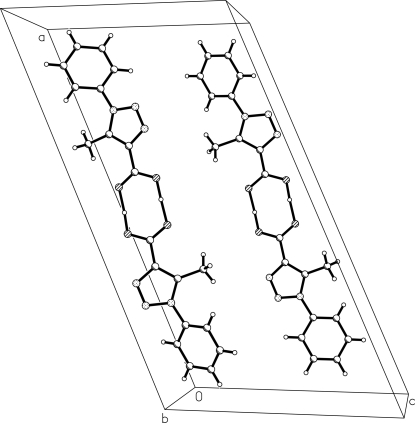
Packing diagram of the title molecules, showing the structure along the b axis.

**Fig. 3.**
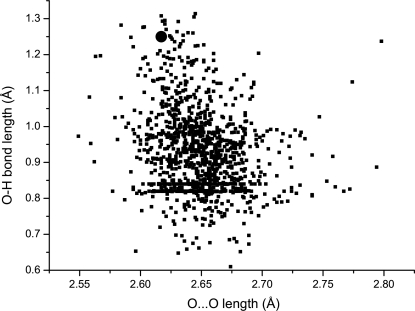
The O-H vs. O···O distances (Å) for the structural motif of the pairs of the carboxyl groups that are interconnected by the hydrogen bonds. The structures were found in the Cambridge Structural Database (Allen, 2002; CSD version 5.29 including the updates from January 25, 2008). The query has been limited for the structures with the R factor <0.05; with no errors, no disorder. The polymers and the powder samples were excluded as well. 1028 hits were found. The title structure is symbolized by the large circle in the left upper corner of the plot.

## supplementary crystallographic information

### Comment

Many triazole-related molecules have received much attention because of their biological activities (Olesen *et al.*, 2003; Tian *et al.*, 2005). We report herein the crystal structure of the title compound (Fig. 1).

The molecules are arranged into inversion dimers *via* carboxyl groups that are interconnected by pairs of the O-H···O hydrogen bonds (Fig. 2). The graph-set motif is R^2^_2_(8) (Etter *et al.*, 1990). The peculiarity of the title structure consists in the displacement of the bonding hydrogen towards the centre of the hydrogen bond (Tab. 1). Though not unprecedented, Fig. 3 shows that the title structure belongs among rather rare examples where in a relatively long O···O hydrogen bond the involved hydrogen is shifted towards the centre. The displacement of the bonding hydrogen towards the centre of the hydrogen bond is concomitant to more equal C-O bonding distances in the carboxyl group.

The dihedral angle between the triazole and phenyl ring planes is 41.85 (1)°.

### Experimental

The title compound was prepared from azidobenzene according to the reported method (El Khadem *et al.*, 1968). The colourless prisms (average size: 0.5×0.8×1.0 mm) were obtained by slow evaporation from 95% ethanol/water solution at room temperature.

### Refinement

All the hydrogen atoms could have been discerned in the difference electron density map, nevertheless, all the H atoms attached to the carbon atoms were constrained in a riding motion approximation. C_aryl_—H=0.93 Å, with *U*_iso_(H)=1.2*U*_eq_(C). C_methyl_—H=0.96 Å, with *U*_iso_(H)=1.5*U*_eq_(C). The hydroxyl hydrogen was refined freely.

### Crystal data

**Table:**

C_10_H_9_N_3_O_2_	*F*_000_ = 848
*M**_r_* = 203.20	*D*_x_ = 1.278 Mg m^−^^3^
Monoclinic, *C*2/*c*	Mo *K*α radiation λ = 0.71073 Å
Hall symbol: -C 2yc	Cell parameters from 2025 reflections
*a* = 23.616 (3) Å	θ = 2.8–27.5º
*b* = 7.7189 (15) Å	µ = 0.09 mm^−^^1^
*c* = 12.606 (2) Å	*T* = 293 (2) K
β = 113.18 (3)º	Prism, colourless
*V* = 2112.5 (8) Å^3^	0.20 × 0.18 × 0.15 mm
*Z* = 8	

### Data collection

**Table:**

Rigaku SCXmini diffractometer	2400 independent reflections
Radiation source: fine-focus sealed tube	1583 reflections with *I* > 2σ(*I*)
Monochromator: graphite	*R*_int_ = 0.053
*T* = 293(2) K	θ_max_ = 27.5º
ω scans	θ_min_ = 2.8º
Absorption correction: multi-scan(CrystalClear; Rigaku, 2005)	*h* = −30→30
*T*_min_ = 0.965, *T*_max_ = 0.977	*k* = −9→10
10370 measured reflections	*l* = −16→16

### Refinement

**Table:**

Refinement on *F*^2^	Secondary atom site location: difference Fourier map
Least-squares matrix: full	Hydrogen site location: difference Fourier map
*R*[*F*^2^ > 2σ(*F*^2^)] = 0.062	H atoms treated by a mixture of independent and constrained refinement
*wR*(*F*^2^) = 0.148	*w* = 1/[σ^2^(*F*_o_^2^) + (0.0614*P*)^2^ + 0.4952*P*] where *P* = (*F*_o_^2^ + 2*F*_c_^2^)/3
*S* = 1.08	(Δ/σ)_max_ < 0.001
2400 reflections	Δρ_max_ = 0.14 e Å^−^^3^
141 parameters	Δρ_min_ = −0.18 e Å^−^^3^
31 constraints	Extinction correction: none
Primary atom site location: structure-invariant direct methods	

### Special details

**Table:**

Geometry. All e.s.d.'s (except the e.s.d. in the dihedral angle between two l.s. planes) are estimated using the full covariance matrix. The cell e.s.d.'s are taken into account individually in the estimation of e.s.d.'s in distances, angles and torsion angles; correlations between e.s.d.'s in cell parameters are only used when they are defined by crystal symmetry. An approximate (isotropic) treatment of cell e.s.d.'s is used for estimating e.s.d.'s involving l.s. planes.
Refinement. Refinement of *F*^2^ against ALL reflections. The weighted *R*-factor *wR* and goodness of fit *S* are based on *F*^2^, conventional *R*-factors *R* are based on *F*, with *F* set to zero for negative *F*^2^. The threshold expression of *F*^2^ > σ(*F*^2^) is used only for calculating *R*-factors(gt) *etc*. and is not relevant to the choice of reflections for refinement. *R*-factors based on *F*^2^ are statistically about twice as large as those based on *F*, and *R*- factors based on ALL data will be even larger.

### Fractional atomic coordinates and isotropic or equivalent isotropic displacement parameters (Å^2^)

**Table:**

	*x*	*y*	*z*	*U*_iso_*/*U*_eq_	
C1	0.41473 (10)	0.1278 (3)	0.72134 (18)	0.0555 (5)	
C2	0.34886 (9)	0.1172 (3)	0.69727 (16)	0.0491 (5)	
C3	0.31868 (9)	0.1493 (2)	0.76949 (15)	0.0461 (5)	
C4	0.20319 (9)	0.1379 (2)	0.72119 (16)	0.0466 (5)	
C5	0.20037 (10)	0.0713 (3)	0.82132 (17)	0.0538 (5)	
H5	0.2350	0.0218	0.8780	0.065*	
C6	0.14517 (11)	0.0799 (3)	0.8352 (2)	0.0655 (6)	
H6	0.1426	0.0364	0.9020	0.079*	
C7	0.09414 (11)	0.1525 (3)	0.7506 (2)	0.0761 (7)	
H7	0.0571	0.1574	0.7603	0.091*	
C8	0.09745 (10)	0.2184 (3)	0.6509 (2)	0.0734 (7)	
H8	0.0626	0.2666	0.5940	0.088*	
C9	0.15210 (9)	0.2129 (3)	0.63569 (18)	0.0578 (5)	
H9	0.1547	0.2585	0.5695	0.069*	
C10	0.34155 (10)	0.2068 (3)	0.89199 (16)	0.0579 (6)	
H10A	0.3099	0.2716	0.9041	0.087*	
H10B	0.3773	0.2786	0.9091	0.087*	
H10C	0.3522	0.1073	0.9417	0.087*	
N1	0.30730 (8)	0.0743 (2)	0.58988 (14)	0.0577 (5)	
N2	0.25230 (8)	0.0763 (2)	0.59058 (13)	0.0571 (5)	
N3	0.25867 (7)	0.1228 (2)	0.70044 (13)	0.0475 (4)	
O1	0.45271 (7)	0.1465 (2)	0.82345 (13)	0.0733 (5)	
O2	0.43008 (7)	0.1161 (3)	0.63484 (13)	0.0799 (6)	
H2	0.4867 (19)	0.125 (5)	0.661 (3)	0.193 (17)*	

### Atomic displacement parameters (Å^2^)

**Table:**

	*U*^11^	*U*^22^	*U*^33^	*U*^12^	*U*^13^	*U*^23^
C1	0.0557 (12)	0.0670 (14)	0.0444 (12)	0.0033 (10)	0.0201 (10)	0.0024 (10)
C2	0.0508 (11)	0.0572 (12)	0.0404 (11)	0.0029 (9)	0.0190 (9)	0.0016 (8)
C3	0.0505 (11)	0.0472 (11)	0.0407 (10)	0.0013 (8)	0.0182 (9)	0.0020 (8)
C4	0.0488 (10)	0.0506 (11)	0.0416 (11)	−0.0023 (9)	0.0190 (9)	−0.0050 (8)
C5	0.0562 (12)	0.0617 (13)	0.0443 (11)	−0.0008 (9)	0.0206 (10)	−0.0031 (9)
C6	0.0660 (14)	0.0826 (16)	0.0573 (14)	−0.0081 (12)	0.0343 (12)	−0.0071 (11)
C7	0.0548 (14)	0.102 (2)	0.0797 (17)	−0.0006 (13)	0.0350 (14)	−0.0096 (15)
C8	0.0528 (13)	0.0884 (18)	0.0717 (16)	0.0117 (12)	0.0165 (12)	0.0013 (13)
C9	0.0566 (13)	0.0654 (14)	0.0481 (12)	0.0046 (10)	0.0169 (10)	0.0026 (10)
C10	0.0580 (12)	0.0714 (15)	0.0429 (11)	−0.0024 (10)	0.0184 (10)	−0.0071 (10)
N1	0.0544 (10)	0.0770 (12)	0.0430 (10)	0.0019 (9)	0.0208 (8)	−0.0018 (8)
N2	0.0567 (11)	0.0774 (13)	0.0381 (9)	−0.0015 (8)	0.0196 (8)	−0.0064 (8)
N3	0.0505 (9)	0.0554 (10)	0.0372 (8)	0.0005 (7)	0.0178 (7)	−0.0002 (7)
O1	0.0532 (9)	0.1133 (14)	0.0506 (9)	−0.0020 (8)	0.0176 (8)	−0.0084 (8)
O2	0.0585 (10)	0.1372 (16)	0.0504 (9)	0.0041 (9)	0.0282 (8)	0.0032 (9)

### Geometric parameters (Å, °)

**Table:**

C1—O1	1.254 (2)	C6—H6	0.9300
C1—O2	1.281 (2)	C7—C8	1.386 (3)
C1—C2	1.465 (3)	C7—H7	0.9300
C2—N1	1.364 (3)	C8—C9	1.378 (3)
C2—C3	1.382 (3)	C8—H8	0.9300
C3—N3	1.356 (2)	C9—H9	0.9300
C3—C10	1.489 (3)	C10—H10A	0.9600
C4—C5	1.389 (3)	C10—H10B	0.9600
C4—C9	1.389 (3)	C10—H10C	0.9600
C4—N3	1.438 (2)	N1—N2	1.302 (2)
C5—C6	1.384 (3)	N2—N3	1.380 (2)
C5—H5	0.9300	O2—H2	1.25 (4)
C6—C7	1.376 (3)		
			
O1—C1—O2	123.66 (19)	C8—C7—H7	119.7
O1—C1—C2	119.41 (19)	C9—C8—C7	120.3 (2)
O2—C1—C2	116.94 (19)	C9—C8—H8	119.8
N1—C2—C3	109.96 (17)	C7—C8—H8	119.8
N1—C2—C1	120.64 (17)	C8—C9—C4	118.7 (2)
C3—C2—C1	129.38 (19)	C8—C9—H9	120.7
N3—C3—C2	103.29 (16)	C4—C9—H9	120.7
N3—C3—C10	124.88 (17)	C3—C10—H10A	109.5
C2—C3—C10	131.79 (18)	C3—C10—H10B	109.5
C5—C4—C9	121.47 (19)	H10A—C10—H10B	109.5
C5—C4—N3	120.56 (18)	C3—C10—H10C	109.5
C9—C4—N3	117.90 (17)	H10A—C10—H10C	109.5
C6—C5—C4	118.8 (2)	H10B—C10—H10C	109.5
C6—C5—H5	120.6	N2—N1—C2	108.68 (15)
C4—C5—H5	120.6	N1—N2—N3	107.15 (15)
C7—C6—C5	120.2 (2)	C3—N3—N2	110.92 (15)
C7—C6—H6	119.9	C3—N3—C4	131.92 (16)
C5—C6—H6	119.9	N2—N3—C4	117.15 (15)
C6—C7—C8	120.5 (2)	C1—O2—H2	113.8 (15)
C6—C7—H7	119.7		

### Hydrogen-bond geometry (Å, °)

**Table:**

*D*—H···*A*	*D*—H	H···*A*	*D*···*A*	*D*—H···*A*
O2—H2···O1^i^	1.25 (4)	1.38 (4)	2.617 (2)	173 (3)

Symmetry codes: (i) −*x*+1, *y*, −*z*+3/2.
